# Estimated age and gender profile of individuals missed by a home-based HIV testing and counselling campaign in a Botswana community

**DOI:** 10.7448/IAS.18.1.19918

**Published:** 2015-05-29

**Authors:** Vlad Novitsky, Hermann Bussmann, Lillian Okui, Andrew Logan, Sikhulile Moyo, Erik van Widenfelt, Mompati Mmalane, Quanhong Lei, Molly P Holme, Joseph Makhema, Shahin Lockman, Victor Degruttola, M Essex

**Affiliations:** 1 Department of Immunology and Infectious Diseases, Harvard School of Public Health Boston, MA, USA; 2 Botswana–Harvard AIDS Institute Partnership, Gaborone, Botswana

**Keywords:** HIV-1, home-based HTC, Botswana, gender, age, missing individuals, individuals tested for the first time

## Abstract

**Introduction:**

It would be useful to understand which populations are not reached by home-based HIV-1 testing and counselling (HTC) to improve strategies aimed at linking these individuals to care and reducing rates of onward HIV transmission.

**Methods:**

We present the results of a baseline home-based HTC (HBHTC) campaign aimed at counselling and testing residents aged 16 to 64 for HIV in the north-eastern sector of Mochudi, a community in Botswana with about 44,000 inhabitants. Collected data were compared with population references for Botswana, the United Nations (UN) estimates based on the National Census data and the Botswana AIDS Impact Survey IV (BAIS-IV). Analyzed data and references were stratified by age and gender.

**Results:**

A total of 6238 age-eligible residents were tested for HIV-1; 1247 (20.0%; 95% CI 19.0 to 21.0%) were found to be HIV positive (23.7% of women vs. 13.4% of men). HIV-1 prevalence peaked at 44% in 35- to 39-year-old women and 32% in 40- to 44-year-old men. A lower HIV prevalence rate, 10.9% (95% CI 9.5 to 12.5%), was found among individuals tested for the first time. A significant gender gap was evident in all analyzed subsets. The existing HIV transmission network was analyzed by combining phylogenetic mapping and household structure. Between 62.4 and 71.8% of all HIV-positive individuals had detectable virus. When compared with the UN and BAIS-IV estimates, the proportion of men missed by the testing campaign (48.5%; 95% CI 47.0 to 50.0%) was significantly higher than the proportion of missed women (14.2%; 95% CI 13.2 to 15.3%; *p*<0.0001). The estimated proportion of missed men peaked at about 60% in the age group 30 to 39 years old. The proportions of missed women were substantially smaller, at approximately 28% within the age groups 30 to 34 and 45 to 49 years old.

**Conclusions:**

The HBHTC campaign seems to be an efficient tool for reaching individuals who have never been tested previously in southern African communities. However, about half of men from 16 to 64 years old were not reached by the HBHTC, including about 60% of men between 30 and 40 years old. Alternative HTC strategies should be developed to bring these men to care, which will contribute to reduction of HIV incidence in communities.

## Introduction

The HIV-1 prevalence rate in many southern African countries remains high and exceeds 15% in the general population [1–4]. The introduction and scale-up of antiretroviral treatment (ART) programmes have led to both substantial increases in life expectancy and reduction of HIV transmissions [5–15]. However, ART can only be initiated in those HIV-positive individuals who have been tested and linked to health care. Knowledge of one's HIV status can also help to reduce high-risk transmission behaviour [16–18].

A substantial fraction of HIV-positive individuals in southern Africa are still not aware of their HIV status. Despite decreasing proportions of individuals with unknown HIV status over the last decade, the prevalence of undiagnosed HIV infection in sub-Saharan Africa remains high [19–25]. In southern Africa, a smaller proportion of men than of women are aware of their HIV status [26–29]. HIV transmission rates are substantially higher from undiagnosed individuals [30]. The more widely ART is used, the greater the likelihood that HIV-positive individuals who are unaware of their HIV status will become the major source of onward HIV transmission. To be successful, ART-based HIV prevention strategies need to identify and engage people who are not aware of their HIV infection by expanding HIV testing and counselling (HTC) [31–33].

The diversification of approaches for HTC could help to overcome fear and stigma associated with HIV testing at health-care facilities. Home-based HTC (HBHTC) is a valuable approach that could improve the diagnostics of HIV and facilitate linkage to health care; it has been successfully used in many sub-Saharan African countries [34–47]. The success of HBHTC has led to the design and implementation of a series of clinical trials in southern Africa with enhanced HBHTC as a key intervention strategy [48–51].

The purpose of this study was to investigate the HIV-1 prevalence in a peri-urban community in Botswana and to use the inferred population structure to estimate age and gender profiles for individuals who were not reached and tested during the HBHTC campaigns. The main rationale for the study was the need to better understand what populations are not being reached by HBHTC and to learn about their structure. Knowledge gained could help in developing targeted prevention interventions and improving linkage to care. Data were analyzed under two different assumptions about the distributions of age and gender among individuals not tested. The first assumption is that *tested* and *untested* individuals have similar population structures. The second assumption is that only the population of individuals *tested for the first time* (a subset of tested individuals) has the same structure as the population of individuals who were not tested during HBHTC.

## Methods

### Human subjects

The study was approved by IRBs in Botswana and at the Harvard School of Public Health. All participants provided written informed consent (or assent with guardian permission, in the case of persons 16 to less than 18 years of age).

### Study design

The Mochudi Prevention Project (MPP) has been previously described [52]. The MPP was performed as an open cohort community-based study that measured uptake of repeated approximately annual HIV testing, questionnaires, behavioural prevention messages and referrals for ART or male circumcision through free Botswanan government programmes. Three rounds of HBHTC were conducted in one sector of a village in Botswana (Mochudi) to estimate HIV-1 incidence and prevalence among 16- to 64-year-old residents over time. Only unique data from the first (enrolment) visit were used in this study. To avoid overlaps, the repeated household visits were not included. Community engagement activities, consenting, HIV testing, counselling and data/sample collection were conducted during the period May 2010 to August 2013.

### Study subjects

**Figure 1:**
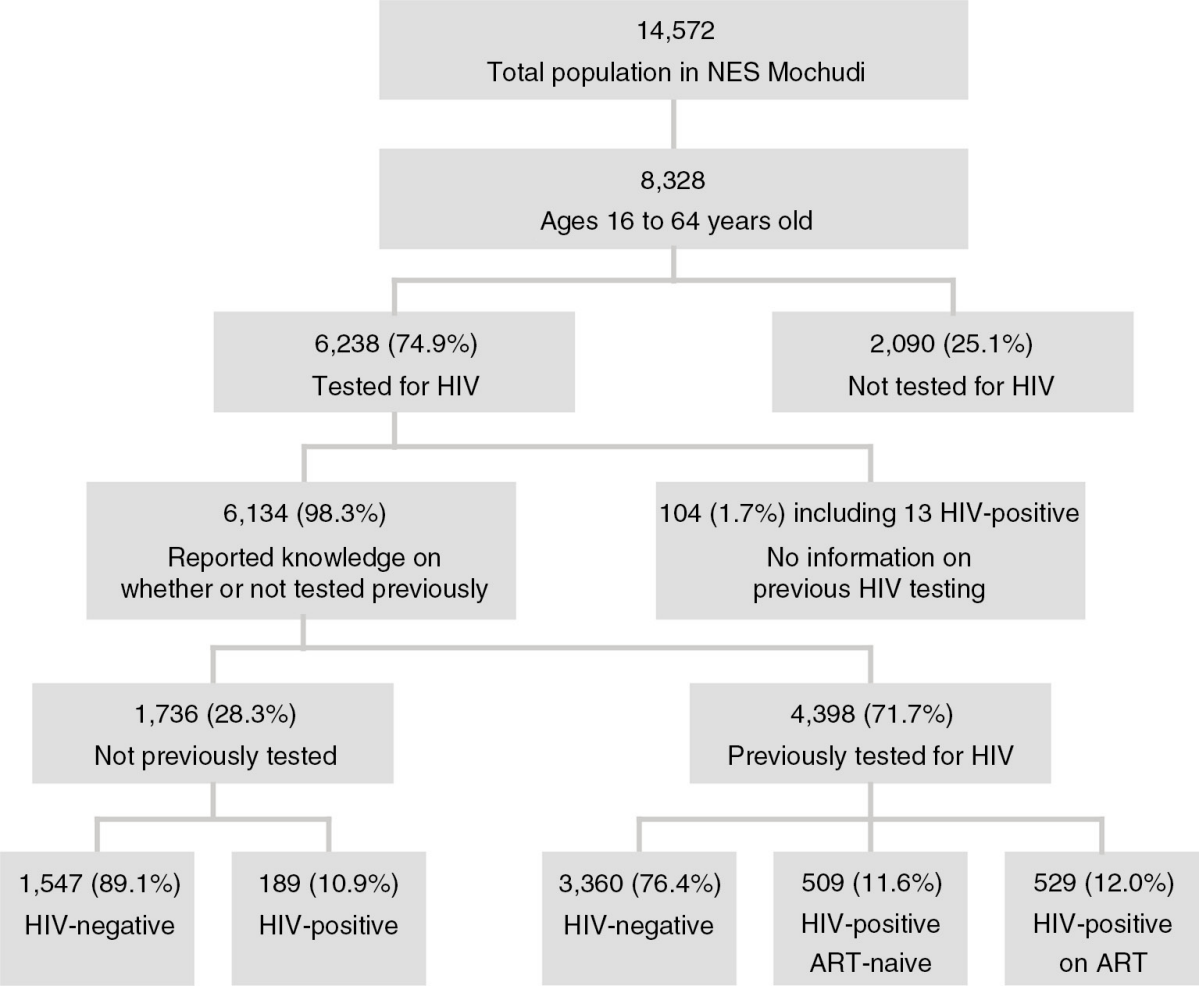
Flowchart of home-based HIV-1 testing and counselling in the north-east segment of Mochudi.

Based on Botswanan census data from 2011 [53], the estimated total population in Mochudi was 44,339. The MPP survey covered the north-eastern sector of Mochudi, which is a geographically distinct area of the village separated by a river and a hill from remaining parts of Mochudi. The MPP survey enumerated 3650 households with 14,572 residents. A total of 8328 enumerated residents were within the age range of 16 to 64 years old and eligible for the study (Figure 1).

### Procedures during household visits

During the household visits, eligible residents were asked to complete an individual questionnaire with socio-demographic and HIV-related information, including a history of their HIV testing, their ART status and patterns of sexual behaviour, and to donate a blood sample for a rapid HIV test. Capillary blood samples were collected and stored as dried blood spots (DBSs) for viral load test and viral genotyping (if HIV positive). HIV-positive individuals were referred to the Botswanan national ART programme (free-of-charge treatment of all adults with CD4 ≤350 cells/µL or WHO Stage 3/4). ART-naïve HIV-positive individuals (newly diagnosed or linked to care) were invited to a clinic to determine their eligibility for initiation of ART. A clinic visit included collection of venous blood by phlebotomy for CD4 and HIV-1 RNA testing.

### HIV-1 testing

HIV-1 testing was performed in the household using Botswanan HIV testing guidelines and running two rapid tests in parallel: Determine HIV-1/2 (Abbott Laboratories, Chicago, IL, USA) and Uni-Gold™ (Trinity Biotech, Wicklow, Ireland). Only concordant results in both tests were considered valid. If results were discordant, the participant was invited to a clinic and blood was collected for confirmatory HIV testing, performed at a reference laboratory using double EIA (Murex HIV 1.2.O test, Murex Biotech Ltd., Dartford, UK, and Vironostika Uni-Form II plus 0 EIA, BioMerieux, Durham, NC, USA) and/or Western blot (Genetic Systems HIV-1 Western Blot, Bio-Rad Laboratories, Redmond, WA, USA). The results of EIA and/or Western blot superseded the discordant or indeterminate results obtained in the field.

### HIV-1 RNA load testing

During the clinic visit for assessment of eligibility for initiation of ART, venous blood was collected by phlebotomy in ART-naïve HIV-positive individuals. DBSs were collected during HBHTC from individuals on ART. The HIV-1 RNA load was quantified by Roche AmpliPrep/AMPLICOR MONITOR, version 1.5 (Mannheim, Germany), or Abbott m2000sp/Abbott m2000rt (Wiesbaden, Germany), at the Botswana–Harvard HIV Reference Laboratory (BHHRL), which was accredited by the South African National Accreditation System for HIV-1 viral load testing [54]. BHHRL maintains certification in Rush University's Virology Quality Assurance Program [55]. In this study, HIV-1 RNA load above 400 cps/ml was considered detectable.

### Population references

Two estimates of population in Botswana were utilized in the study, with categories defined by five-year age groupings and gender. These were derived from the United Nations DESA, Population Division, based on Botswanan census data [56], and the Botswana AIDS Impact Survey IV (BAIS-IV) [57]. The numbers of individuals tested for HIV in Mochudi were compared with each population reference. In some analyses, the numbers of tested individuals in Mochudi were compared with the average values between the UN and BAIS-IV references. All comparisons were performed per five-year age group and separately by gender. The difference between the number of tested individuals and the reference number (per age group and gender) was interpreted as the estimated number of non-tested individuals within the corresponding age group and gender.

### HIV-1C *env* gp120 sequences

The HIV-1C *env* gp120 V1C5 sequences were generated by population-based (bulk) Sanger sequencing, as described previously [52]. A single sequence per subject was used in this study. The conserved and variable regions corresponding to functional domains within HIV-1 *env* gp120 were aligned separately by applying differential penalties for gap opening and gap extension. HIV cluster was defined as a viral lineage that gives rise to a monophyletic sub-tree of the overall phylogeny with strong statistical support. In the context of high sampling density, phylogenetically distinct viral lineages are likely to represent HIV transmission chains. HIV clusters were identified by a combination of bootstrapped maximum likelihood ≥0.80 [58–60] and internode certainty ≥0.70 [61, 62]. Clusters with internode certainty between 0.50 and 0.70 were also considered. As we demonstrated recently, HIV clusters identified by bootstrapped maximum likelihood had low intracluster distances [52].

### Statistical analysis

All confidence intervals of estimated proportions are asymptotic 95% binomial confidence intervals (95% CI) computed with the prop.test function in R version 3.0.1 [63]. Comparisons between proportions were performed using a two-sample test for equality of proportions with continuity correction (chi-squared test statistic). Two-sample Kolmogorov-Smirnov test (ks.test) was used for comparison of age distributions. *P* values less than 0.05 were considered statistically significant and all hypothesis tests were two-sided. Statistical analysis was performed using R version 3.0.1. Plots and histograms were produced in R. All figures were finalized in Adobe Illustrator CS6.

## Results

### HIV prevalence

The results of HIV testing and accompanying age and gender information were available for 6238 of 8328 (74.9%; 95% CI 74.0 to 75.8%) age-eligible residents. The estimated proportion of HIV-positive individuals was 20.0% (95% CI 19.0 to 21.0%) with a substantial gender gap, 13.4% (95% CI 12.0 to 14.8%) among men and 23.7% (95% CI 22.4 to 25.1%) among women (*p* value for comparison between proportion of HIV-positive men and women is <0.00001; two-sample test for equality of proportions with continuity correction). Using the United Nations DoEaSA age proportion data [56], the overall age-adjusted HIV-1 prevalence was 18.8% (95% CI 17.8 to 19.8%). The age-adjusted HIV prevalence was 14.3% (95% CI 12.7 to 15.9%) among men and 23.4% (95% CI 21.9 to 24.9%) among women.

**Figure 2:**
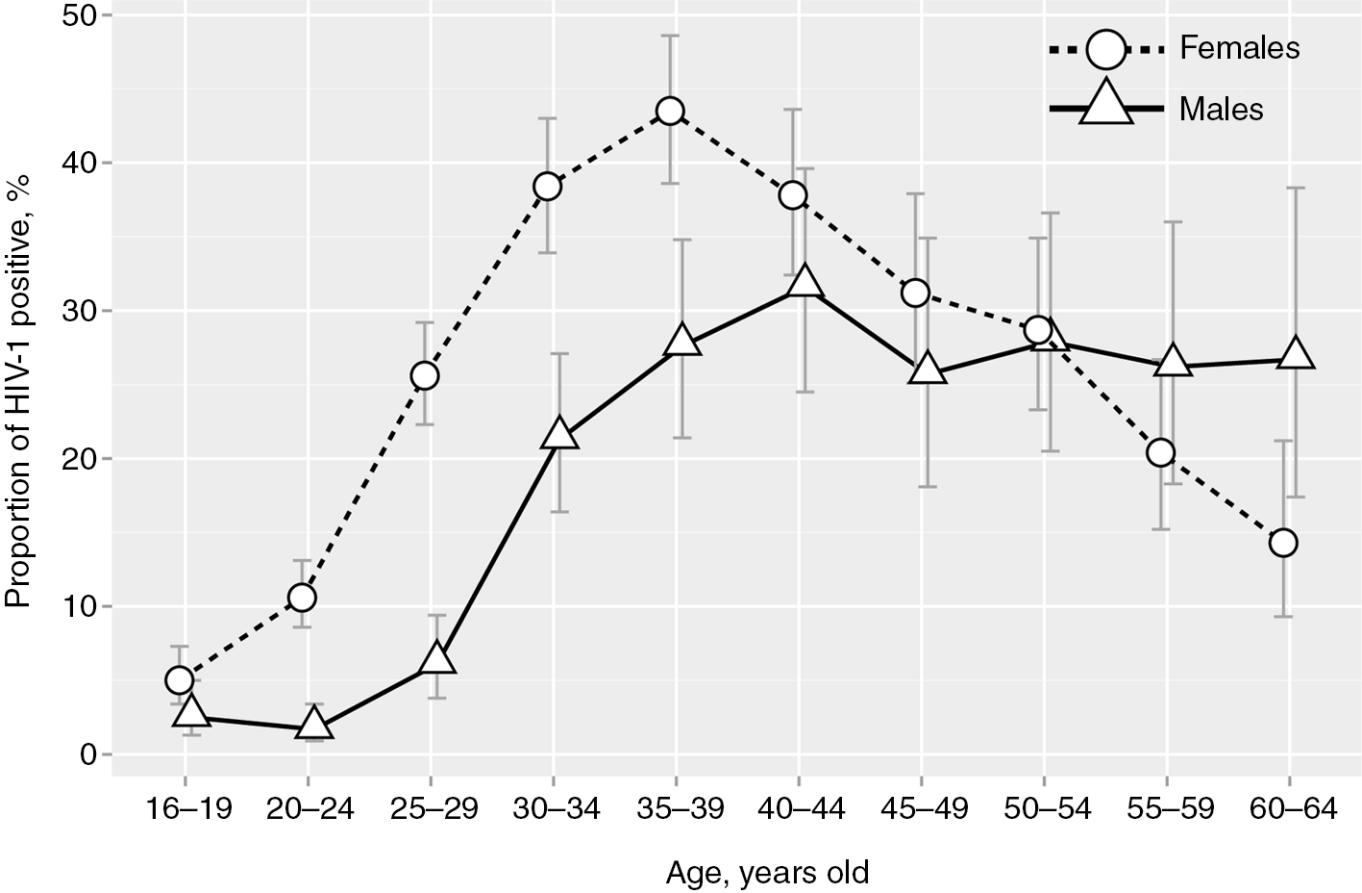
Proportion of HIV-positive individuals among adults tested in the north-east segment of Mochudi stratified by age and gender. Females are indicated by circles and dashed lines. Males are depicted by triangles and solid lines. Error bars indicate 95% confidence intervals.

Among those tested in Mochudi, HIV-1 infection differed by age group, with an earlier rise in women than in men (Figure 2). A sharp increase in HIV-1 prevalence occurred in young women about a decade earlier than it did in men. A gradual increase in the proportion of HIV-positive women up to the late thirties was followed by a gradual decrease to 14.3% in the age group 60 to 64 years old. HIV-1 prevalence among women peaked at 43.5% (95% CI 38.6 to 48.6%) in the age group 35 to 39 years. The proportion of HIV-positive men remained under 10% until age 29, and then gradually increased to 21, 28 and 32% in the age groups 30 to 34, 35 to 39, and 40 to 44 years, respectively. In men over 44 years old, HIV-1 prevalence plateaued at the level of 26-27%.

HIV-1 prevalence was higher in women than in men within each five-year age category between 20 and 39 years (*p*<0.001). In the oldest age group (60 to 64 years), this pattern was reversed and it was higher in men (*p*=0.039). No significant difference in HIV-1 prevalence was observed within the youngest age group (16 to 19 years old) or in age groups from 40 to 59 years (*p*>0.05).

### Combined phylogeny and HBHTC data

The HIV-1C V1C5 sequences and household structure data were available for a subset of 833 subjects from 677 households in Mochudi. Of those, 322 (38.7%; 95% CI 35.3 to 42.1%) subjects were found in clusters, and 511 (61.3%; 95% CI 57.9 to 64.7%) were not.

To better understand patterns of HIV transmission in Mochudi, a combined analysis using phylogenetic mapping and household structure was performed. Households with available HIV sequencing data were grouped into households with a single HIV-positive individual and those with multiple HIV-positive individuals. To assess whether the extent of HIV clustering differed between individuals residing in households with a single versus with multiple HIV-positive individuals, proportions of clustered individuals in these groups of households were compared. The majority of individuals, 549 of 833 (65.9%; 95% CI 62.6 to 69.1%), resided in households with a single HIV-positive person. About one-third of them, 187 of 549 (34.1%; 95% CI 30.1 to 38.2%), were found in clusters, and 362 were not in clusters. Two hundred and eighty-four participants resided in the 128 households with multiple HIV-positive individuals. Of those participants, 135 (47.5%; 95% CI 41.6 to 53.5%) were found in clusters and 149 (52.5%; 95% CI 46.5 to 58.4%) not in clusters. Analysis of proportions revealed that individuals residing in households with multiple HIV-positive individuals were more likely to be in clusters than individuals from households with a single HIV-positive person (*p*<0.001, Fisher exact test).

To analyze whether individuals from the same household cluster together, phylogenetic analysis was used. The analysis cannot address directionality of HIV transmission on an individual level, to resolve the issue of “who infected whom.” However, on a population level, clustering of HIV-positive individuals suggests that these individuals are likely to share the same HIV transmission chain. The converse is not true: genotypes from the same chain may not cluster due to the absence of samples from people whose position in the transmission chain is between those with observed sequences. This notion is critical for the interpretation of clustering results for HIV-positive individuals residing in the same household. Among 128 households with multiple HIV-positive individuals, participants residing in only 16 (12.5%; 95% CI 7.5 to 19.8%) households clustered together with another HIV-positive person from the same household. Male/female pairs were found in 15 of 16 households, while in one case the pair included two women (a 40-year old mother and her 18-year old daughter).

Lack of clustering can be equally informative, as it suggests that household residents are infected with phylogenetically distinct HIV lineages and are not likely to be in the same transmission chain. The majority of households with multiple HIV-positive individuals, 116 of 128 (90.6%; 95% CI 83.9 to 94.8%), had phylogenetically distinct HIV transmission chains. This number includes four households with two individuals infected through the same transmission chain, and another HIV-positive person infected through a different transmission chain. In 45 households, all HIV-positive residents were found outside of clusters, suggesting HIV infection with phylogenetically unique lineages that did not circulate in Mochudi (or were not detected in this study). In 21 households, residents were found in clusters with individuals from other households, suggesting infection with locally circulating HIV lineages. In 50 households, a mix of non-clustered individuals and those who clustered with individuals from other households was found.

To evaluate the spread of HIV lineages in Mochudi, we assessed whether identified HIV lineages were shared between households. If so, HIV-positive individuals in these households are likely to share viral transmission chains. We estimated proportions of clustered individuals based on whether they clustered with individuals from the same household or a different one. Among 322 clustered subjects, only 32 (9.9%; 95% CI 7.0 to 13.9%) clustered within the same household. The vast majority of clustered subjects, 290 of 322 (90.1%; 95% CI 86.1 to 93.0%), were found in clusters with subjects from other households, suggesting high complexity within the local HIV transmission network in Mochudi.

Clustered individuals seemed to be younger than non-clustered individuals. This notion was supported by a statistically significant difference in age distribution between clustered and non-clustered females (*p*=0.00012, two-sample Kolmogorov-Smirnov test). However, the difference in age distribution between clustered and non-clustered males did not reach statistical significance (*p*>0.1, two-sample Kolmogorov-Smirnov test).

### Individuals tested for the first time in the HBHTC campaigns

Participants were asked about their history of HIV testing. After excluding 529 individuals on ART, a total of 5605 ART-naïve residents with available gender and age data reported the history of their previous HIV testing, if any (Figure 1; 6134 individuals with a history of HIV testing minus 529 individuals on ART=5605 individuals). Among ART-naïve individuals, 1736 (31.0%; 95% CI 29.8 to 32.2%) reported that they had never been tested previously, and 189 (10.9%; 95% CI 9.5 to 12.5%) of them tested HIV positive during the HBHTC. A gender difference was evident in HIV prevalence among individuals tested for the first time. The proportion of HIV-positive men among new testers, at 8.0% (95% CI 6.2 to 10.2%), was significantly lower than that of women, at 13.1% (95% CI 11.1 to 15.4%), *p*=0.0008 (two-sample test for equality of proportions with continuity correction). A total of 3869 ART-naïve individuals reported that they had previously been tested for HIV (69.0%; 95% CI 67.8 to 70.2%). Among these previously tested subjects, 509 (13.2%; 95% CI 12.1 to 14.3%) tested HIV positive during the HBHTC campaigns. The gender gap was also evident in the subset of previous testers. HIV prevalence among male previous testers was 8.6% (95% CI 7.2 to 10.2%) versus 15.7% (95% CI 14.3 to 17.2%) in female previous testers (*p*<0.00001; two-sample test for equality of proportions with continuity correction). The HIV status and gender distribution for ART-naïve individuals tested for the first time and those tested previously is presented in Table 1. HIV prevalence was higher among those previously tested than those who were being tested for the first time (*p*=0.02; two-sample test for equality of proportions with continuity correction).

**Table 1:** ART-naïve individuals tested for the first time and individuals tested previously

	Tested for the first time			Previously tested		
		
	HIV positive	HIV negative	Total	HIV positive	HIV negative	Total
Both genders	189	1547	1736	509	3360	3869
Males	60	693	753	118	1261	1379
Females	129	854	983	391	2099	2490

**Figure 3:**
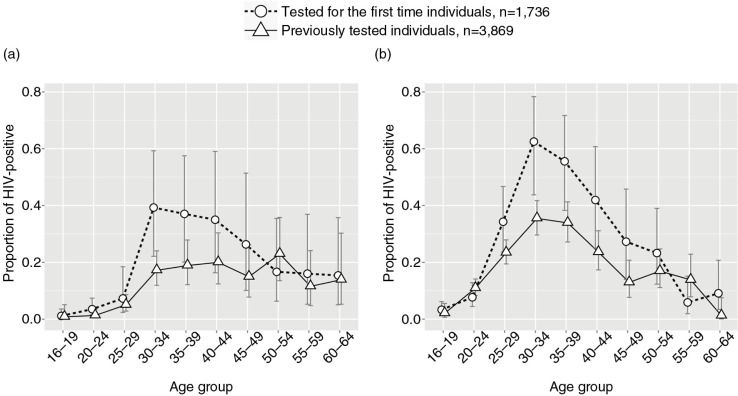
Proportions of HIV positive among ART-naïve individuals tested for the first time (open circles with dashed line) and previously tested (open triangles with solid line). (a) Males. (b) Females. Error bars indicate 95% confidence intervals.

The proportions of HIV-positive individuals were spread unevenly across age groups in both subsets, those tested for the first time and those previously tested (Figure 3). Although HIV prevalence seemed to be higher in middle-aged men (Figure 3a) and women (Figure 3b) tested for the first time, the difference with those previously tested did not reach statistical significance (all *p*>0.05 for all age groups in both genders; two-sample test for equality of proportions with continuity correction).

### HIV cascade of care

**Figure 4:**
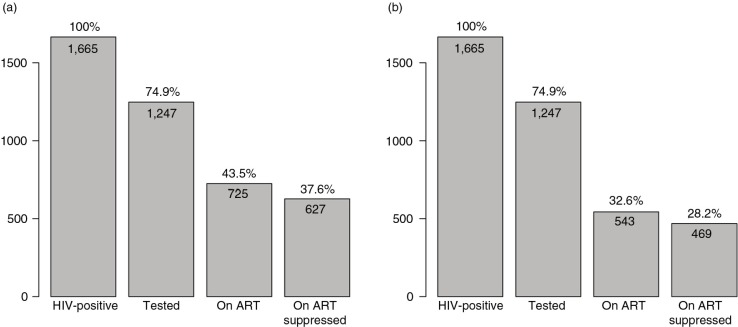
Two scenarios of HIV cascade of care in the north-east segment of Mochudi, Botswana, based on uniform and differential distributions among HIV-positive individuals not tested. Numbers within each bin indicate estimated number of individuals in each category. Percentages above the bins depict the estimated proportion for each category in relation to the total estimated number of HIV-positive individuals in the north-east segment of Mochudi, which is 1665. (a) The assumption that distributions among not tested and tested individuals are similar. (b) The assumption that distributions among not tested individuals are similar to distributions among those tested for the first time.

To estimate the proportions of virologically suppressed (HIV-1 RNA ≤400 cps/ml) and unsuppressed (HIV-1 RNA>400 cps/ml) individuals among HIV-positive individuals in Mochudi, we investigated the local HIV cascade of care. We estimated that the total number of HIV-positive individuals in the NES of Mochudi is *n*=1665. The estimated number was based on the 20.0% prevalence of HIV-1 infection in the NES of Mochudi [52], the number of age-eligible residents enumerated by the MPP and the assumption that HIV prevalence among individuals previously tested and those not tested is similar. Because the true distribution of individuals on ART and virologically suppressed individuals within the subset of individuals not tested was unknown, we explored two possible scenarios. The first scenario is based on the assumption that proportions of individuals on ART and suppressed individuals on ART among HIV-positive individuals not tested and all tested HIV-positive individuals are similar (Figure 4a). According to this assumption, 43.5% (95% CI 41.2 to 46.0%) of all HIV-positive individuals in Mochudi were on ART, and 37.7% (95% CI 35.3 to 40.0%) of all HIV-positive individuals were virologically suppressed. The second scenario is based on an assumption that proportions of individuals on ART and suppressed individuals on ART among HIV-positive individuals not tested is similar to that in a subset of newly diagnosed HIV-positive individuals, i.e. those identified as HIV positive who were tested for the first time (Figure 4b). According to this assumption, a smaller percentage, 32.6% (95% CI 30.1 to 34.9%), of HIV-positive individuals were on ART, and only 28.2% (95% CI 26.0 to 30.4%) of HIV-positive individuals were virologically suppressed. The estimates for the second scenario are based on proportions found within a subset of individuals in the NES of Mochudi who were tested for HIV for the first time. Reciprocally, between 62.4 and 71.8% of all HIV-positive individuals in the NES of Mochudi are virologically unsuppressed, respective to the second and first assumptions.

### Individuals not tested for HIV

**Figure 5:**
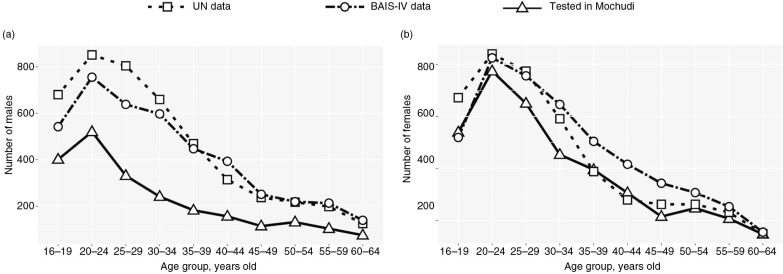
Comparison of individuals tested for HIV during home-based HIV-1 testing and counselling campaigns in Mochudi (triangles with solid lines) with reference estimates based on data from the UN (open squares with dashed lines) and the Botswana AIDS Impact Survey IV (BAIS-IV; open circles with dashed lines). The x-axis shows the age groups. The y-axis denotes the numbers of individuals tested in Mochudi (open triangles connected by solid lines), projected based on the UN data (open squares connected by dashed lines) or based on the BAIS-IV data (open circles connected by dot-dash lines). (a) Males. (b) Females.

To learn more about gender and age distribution among individuals not tested for HIV in the HBHTC campaigns in Mochudi, we utilized the Botswanan population structure (gender and age distributions) reported by the United Nations DoEaSA, Population Division [56] and BAIS-IV [57]. The reference estimates showed relatively similar profiles of age and gender curves for the Botswanan population, with small differences that could be attributed to methodological variations. Whereas the UN data imply slightly higher numbers of young men (Figure 5a, the BAIS-IV estimates imply slightly higher numbers of women within the age groups 30 to 54 years old (Figure 5b). The reference estimates were compared with the numbers of individuals reached by the enhanced HBHTC campaigns in Mochudi per age group for men (Figure 5a) and women (Figure 5b).

The gap between the curves of reference estimates and the number of tested individuals within each age group can be interpreted as “not unreached” or “not tested” individuals. To compare the number of tested individuals with reference estimates, we used the average between the UN and the BAIS-IV data for each age group. We estimated that the number of missed men (both HIV-positive and HIV-negative men not reached by the HBHTC) was 2121, or 48.5% (95% CI 47.0 to 50.0%) of the estimated number of men in the NES of Mochudi. The number of missed women (both HIV-positive and HIV-negative women not reached by the HBHTC) was 652, or 14.2% (95% CI 13.2 to 15.3%) of the estimated number of women in the NES of Mochudi. The difference between the proportions of missed men and missed women was statistically significant (*p*<0.0001).

### Age and gender profiles of individuals not tested

**Figure 6:**
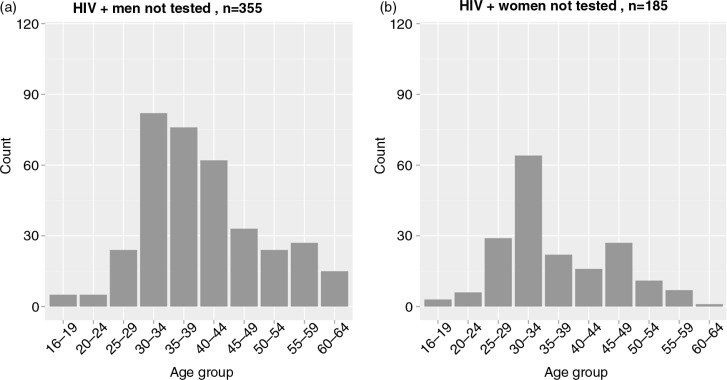
HIV-positive individuals not tested: Estimated distributions stratified by age group and gender according to the first assumption. These estimates are based on the assumption that HIV prevalence among individuals not tested is similar to HIV prevalence among tested individuals.

The uncertainty in this analysis was related to the unknown HIV prevalence among individuals not tested. We considered two different assumptions. The first was that HIV prevalence among individuals not tested is similar to the HIV prevalence among tested individuals. The estimated distributions based on this assumption are presented in Figure 6. The total number of HIV-positive individuals not tested was estimated at 540 (355 men and 185 women). The largest number of men not tested was estimated for the age groups 30 to 34 years old (*n*=82), 35 to 39 years old (*n*=76) and 40 to 44 years old (*n*=62). The largest number of women not tested was estimated for the age group 30 to 34 years old (*n*=64).

**Figure 7:**
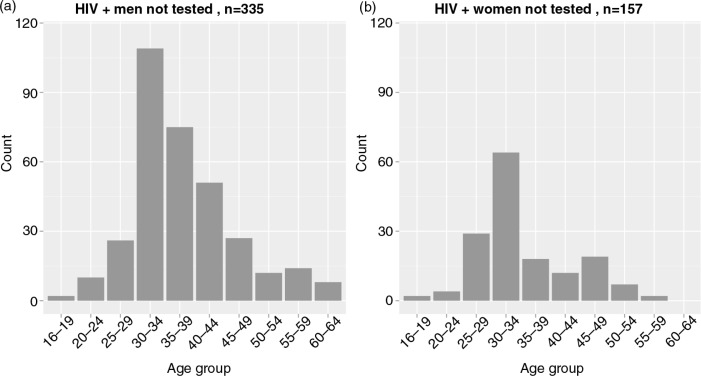
HIV-positive individuals not tested: Estimated distributions stratified by age group and gender according to the second assumption. These estimates are based on the assumption that HIV prevalence among individuals not tested is similar to HIV prevalence among newly diagnosed individuals (tested for the first time).

The second assumption was that HIV prevalence among individuals not tested is similar to HIV prevalence among individuals tested for the first time. The estimated distributions based on the second assumption are presented in Figure 7. The total number of HIV-positive individuals not tested was estimated at 492 (335 men and 157 women). The largest number of men not tested was estimated for the age groups 30 to 34 years old (*n*=109), 35 to 39 years old (*n*=75) and 40 to 44 years old (*n*=51). The largest number of women not tested was estimated for the age group 30 to 34 years old (*n*=64).

**Figure 8:**
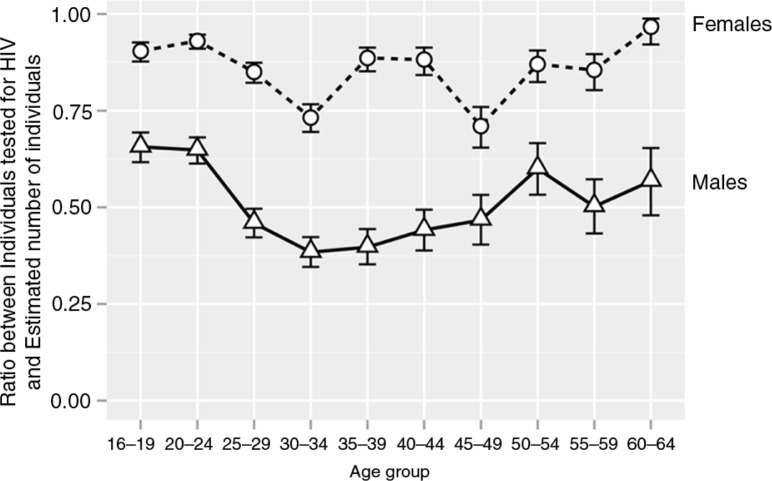
Estimated ratio between individuals tested for HIV and estimated numbers of individuals within specified age group by gender irrespective of their HIV status. Circles connected by dashed line indicate ratios for females. Triangles connected by solid line depict ratios for males. Error bars indicate 95% binomial confidence intervals.

The proportions of individuals tested (not tested) can help to identify, by factors such as age and gender, groups that are the most underrepresented, or below any specified threshold. To assess these proportions, we investigated the proportions according to these factors (Figure 8). For each age group, the proportion of tested women was higher than the proportion of tested men (*p*<0.0001 for comparisons across all age groups; two-sample test for equality of proportions with continuity correction). Middle-aged men from 25 to 49 years old exhibited the lowest proportion (below 50%) of tested men. Reciprocally, the estimated proportion of men not tested peaked at 61.6% for the age group 30 to 34 years old and at 60.3% for the age group 35 to 39 years old. Most proportions of tested women were found at the level of 80 to 85%. The lowest ratios of women tested for HIV were seen for age groups 30 to 34 years old (73.2%) and 45 to 49 years old (71.1%), suggesting that the proportion of women not tested within these age groups was 26.8 and 28.9%, respectively. The curve profiles for the ratio of tested to estimated numbers for the subsets of HIV-negative and HIV-positive individuals were similar to the profiles presented in Figure 8 for all individuals irrespective of HIV status (Supplementary Figure 1). The 95% CIs were broader in the subset of HIV-positive individuals due to smaller numbers.

## Discussion

This study demonstrated the high uptake of community-based household HTC in Mochudi and particularly the high efficiency of household-based HTC in reaching women. The study provides a detailed profile of HIV-1 prevalence by age and gender in this southern African community; it places the focus on the age and gender of those who were missed by the household-based HTC intervention. Analysis of the HIV cascade of care highlighted a high proportion of virologically unsuppressed individuals in the targeted community.

HIV prevalence in 16- to 64-year-olds in Mochudi, a peri-urban village in Botswana, remains high, at 20.0% in 2013. A gender gap in HIV prevalence was evident in the entire population tested for HIV, which included individuals on ART and within the subsets of ART-naïve individuals who had previously been tested or who were being tested for the first time. HIV prevalence among men peaked at 31.6% at ages 40 to 44 years old, whereas HIV prevalence among women peaked at 43.5% at ages 35 to 39 years old.

The higher HIV-1 prevalence among women is consistent with previous reports [27, 41, 64–68] and might plausibly be related to patterns of forming sexual relationships and to higher probability of HIV transmission from males to females. However, the predominantly heterosexual mode of HIV transmission in southern Africa might lead to a smaller-than-observed gender gap in HIV prevalence. A lower HIV prevalence rate among tested men compared to tested women combined with the higher fraction of men not tested raises the question whether the difference in prevalence may result from higher HIV prevalence among men who were not tested compared to those who were.

The combined analysis of phylogenetic mapping and household structure highlighted the complexity of HIV transmission network in Mochudi. Households were grouped by the number of HIV-positive individuals residing in households, single versus multiple. Individuals residing in the households with a single HIV-positive person provided information limited to viral linkage between analyzed households. By contrast, individuals from households with multiple HIV-positive individuals revealed patterns of HIV transmissions both within and between households in this community. We found that in only 12.5% of households with multiple HIV-positive individuals, viral transmission chains were shared between individuals residing in the same household. The vast majority of 322 clustered individuals, about 90%, shared HIV transmission chains with individuals from other households. This finding could be explained in part by the relatively large size of some households and the fact that multiple partner pairs, i.e. families of different generations, reside in the same households. It also highlights the need for cautious interpretation of clustering results from the community in the context of household size.

Taking a closer look at the subset of newly diagnosed individuals is of particular interest, as this group may serve as a bridge to category “individuals not tested,” commonly described as “hard-to-reach” populations [34, 69–75]. Remarkably, more than 30% of individuals tested for HIV during the enhanced HBHTC were tested for the first time, suggesting that HBHTC could be an efficient tool for reaching individuals who have never been tested previously in southern African communities. The difference in HIV prevalence did not reach statistical significance between subsets of individuals tested for the first time and those previously tested.

Our estimates suggest that the majority of HIV-positive individuals in the targeted peri-urban community in Botswana, between 62 and 72%, had detectable virus. These estimates are similar to the distribution of HIV-positive individuals in the HIV cascade of care in the United States [76–78] and Canada [79]. The unsuppressed individuals are the most likely transmitters of HIV, as individuals with low and undetectable HIV-1 RNA load rarely transmit virus [80–82]. The actual distributions (within the HIV cascade of care) within the subset of individuals not tested were unknown, which led us to develop two assumptions for estimating the number of HIV-positive individuals on ART and virologically suppressed individuals on ART. If future studies could reach and test hard-to-reach individuals, our assumptions could be tested.

Knowing the estimated proportions of individuals who are not tested, ART-naïve and unsuppressed while receiving ART could help in developing targeted prevention interventions and improving linkage to care. A targeted approach could be more efficient than a broad scale-up over all categories. For example, if the majority of new HIV transmissions are linked to HIV-positive individuals with unknown HIV status, while individuals not tested comprise less than 30% of all HIV-positive individuals, the enhancement of HTC could be justified as a reasonable programmatic intervention. In contrast, if a relatively small number of individuals with detectable virus on ART are disproportionally associated with a larger proportion of HIV transmissions (e.g. if poor adherence is associated with more risky behaviour), then enhanced monitoring of HIV-1 RNA in individuals on ART could be a justified strategy.

To shed light on the population structure of individuals not tested, we compared individuals reached during the HBHTC campaign with the reference estimates of the population of Botswana. The references were available for the entire Botswanan population, but not for the targeted sector of the peri-urban village of Mochudi. This is a clear limitation of the study that adds uncertainty to our estimates. With this caveat, we estimated that 48.5% of men and 14.2% of women were not reached by the HBHTC campaign in the targeted community. The proportions of missed men peaked in the age group 30 to 39 years old at about 60%, whereas the proportions of missed women peaked at around 28% in the age groups 30 to 34 years old and 45 to 49 years old. This study suggests that HBHTC campaigns seem to be an efficient tool for reaching women for HTC in Botswana. At the same time, the identified age groups of missing men and women necessitates alternative non-standard strategies of HTC in communities, such as mobile voluntary counselling and testing, which was shown to be more successful than HBHTC, particularly among men [19, 83].

Most migration in southern Africa is associated with the search for improved livelihood opportunities, resulting in temporary circular labour migration between rural and urban areas [84–88]. Circular labour migration, or oscillatory movement of young adults, places a heavy burden on rural households, including rapidity of HIV spread [85, 89]. In South Africa, temporary migration levels above 60% for males 30 to 39 years old were reported [85]. In our study, circular labour migration could explain why some men were not reached by household-based HTC. In addition, daily commuting for work, a second home (e.g. at the workplace) and seasonal farming could also contribute to the absence of men in households at the time of HTC. For example, 15% of residents reported spending from 4 to 15 nights per month outside of their household, indicating their high mobility. Among residents normally spending no more than three nights per month outside of their household, 14% reported travelling outside the village from one to three months annually.

These findings collectively highlight the need for developing novel HTC strategies able to target, reach, test and bring to care middle-aged men in communities. A particular focus on mobile men could improve the efficiency of the HTC campaigns [90, 91]. Identifying individuals who are not reached by provider-initiated HTC campaigns, including home-based testing, seems to be central for the success of treatment as prevention programmes and their scale-up.

## Conclusions

The study found high HIV prevalence (about 20%) among 16- to 64-year-old residents in a peri-urban community in Botswana, as well as the presence of a substantial gender gap in HIV prevalence; we estimated the proportion of virologically unsuppressed HIV-positive individuals to be 62 to 72%. The study also describes the gender and age profile of individuals not reached by the HBHTC campaigns in Botswana, to aid in developing targeted prevention interventions. The study suggests that alternative HTC strategies are urgently needed in Botswanan communities.
